# Heterologous microsatellite primers are informative for paca (*Cuniculus paca*), a large rodent with economic and ecological importance

**DOI:** 10.1186/s13104-020-05312-x

**Published:** 2020-10-07

**Authors:** Franco F. Roldán Gallardo, Karen E. DeMatteo, Miguel A. Rinas, Carina F. Argüelles

**Affiliations:** 1 Facultad de Ciencias Exactas, Químicas y Naturales, Departamento de Genética, Universidad Nacional de Misiones (UNaM), Posadas, Misiones Argentina; 2 Grupo de Investigación en Genética Aplicada (GIGA), Instituto de Biología Subtropical (IBS) - Nodo Posadas, UNaM - CONICET, Posadas, Misiones Argentina; 3 Department of Biology & Environmental Studies, Washington University in St. Louis, St. Louis, MO USA; 4 WildCare Institute at the Saint Louis Zoo, St. Louis, MO USA; 5 Ministerio de Ecología y Recursos Naturales Renovables, Posadas, Misiones Argentina

**Keywords:** *Cuniculus paca*, Heterologous amplification, Microsatellite primers, Saliva, Scat

## Abstract

**Objective:**

This study was designed to facilitate genetic studies that would allow information on population structure and genetic diversity of natural or captive stocks of paca (*Cuniculus paca*), a species of ecological and socioeconomic importance, by testing cross-amplification of 20 heterologous microsatellite primer pairs developed for guinea pigs (*Cavia porcellus*) and capybara (*Hydrochoerus hydrochaeris*).

**Results:**

Those primers that showed the best amplification profile in blood samples were subsequently applied to scats and saliva samples, to evaluate their efficiency. Of the 13 microsatellite pairs that amplified in blood, one-third (32%) were successfully amplified in saliva and scat samples. This initial work demonstrates successful cross-amplification in paca providing a solid and promising foundation for future genetic studies with this species. The ability to quantify genetic diversity using noninvasive samples from free-ranging paca is essential to developing applied management strategies for this large neotropical rodent that is not only a prey favored by wide-ranging carnivores [e.g., jaguar (*Panthera onca*), puma (*Puma concolor*)], but is also a species targeted by illegal hunting and wildlife trade.

## Introduction

The paca (*Cuniculus paca*, Linnaeus, 1766), a ground-dwelling, herbivorous rodent, is a unique genus in the family Cuniculidae. The paca is the second largest neotropical rodent with a broad distribution across Central and South America from Mexico to northeast Argentina, with recent introductions in Cuba and the Lesser Antilles [1, 2]. The species inhabits a wide range of humid tropical forests, preferring places near rivers or streams, but can also be found in grasslands, mangrove swamps, and agricultural areas [3]. This medium-sized animal (6-14 kg) is nocturnal, solitary, territorial, monogamous, and sedentary [4]. While it can be vector of zoonotic diseases (e.g., intermediate hosts for *Echinococcus vogeli*; [5]), it is susceptible to others (e.g., leishmaniasis, trypanosomiasis; [2]).

The paca is a favored prey of many carnivores [e.g., coyote (*Canis latrans*), puma (*Puma concolor*), jaguar (*Panthera onca*), bush dog (*Speothos venaticus*)], crocodiles, and heavy-bodied snakes [e.g., boa constrictor (*Boa constrictor*)], among others [2]. Furthermore, it is consumed by human beings, who have targeted this species for its quality meat [6], which is even desired by restaurants [7]. Nevertheless, paca is classified as Least Concerned by the IUCN due to its broad distribution, presumed large populations, and occurrence in protected areas [1]. However, habitat fragmentation or destruction and hunting have led to local extinctions in the southeast portion of its distribution [8]. Consequently, genetic studies are needed to monitor the species’ status, since directed conservation efforts may be needed to ensure their long-term survival.

Genetic analyses with molecular markers are needed to reveal genetic variability at the species and individual level; however, no species-specific microsatellite primers have been developed for the paca. Microsatellites or STRs /SSRs (Short Tandem Repeats / Single Sequence Repeats) are one of the most widely and versatile genetic markers used in molecular ecology to address questions about the conservation and management of threatened and endangered species [9, 10]. The ability to use these markers on noninvasive samples (e.g., scat) removes the need to capture animals directly (e.g., live traps) or indirectly (e.g., camera traps) and focuses on evidence left behind on the animal’s daily movements [11, 12]. While some believe primers designed for a particular species may not be able to amplify the same loci in other phylogenetically-related individuals unless the flanking regions, where the primers hybridize, are highly conserved [13, 14], others suggest this can occur more often than initially thought [15–18].

The ability to use heterologous primers developed for a closely related species has removed limits when species-specific primers are lacking and allowed researchers to quickly expand studies to new species [19, 20]. Cross-amplification has been applied several times at the interface of domestic and free-ranging animals, including the application of domestic cat microsatellite primers to wild cats [11], canid-specific gender identification primers to bush dogs [21, 22], and domestic pig microsatellite primers to wild peccary [23].

Given the lack of species-specific primers for paca, this study drew 20 primers from 34 heterologous primers developed for the superfamily Caviodidea, which the paca shares with guinea pigs (*Cavia porcellus*; [24]) and capybara (*Hydrochoerus hydrochaeris*; [25, 26]). The 20 evaluated primer pairs were selected because they had an expected amplicon size of < 300 bp (range 106–289 bp; [24, 26]). While initial testing of cross-amplification would be done with invasive samples (e.g., blood, saliva), the long-term goal is to have the selected primers work with noninvasive samples or forensic DNA from free-ranging populations, including in the biological corridor proposed for northern-central zone of Misiones, Argentina [27].

## Main text

### Methods

#### Sample collection

Blood, buccal swabs, and scats samples were obtained from three individual paca held at the breeding center of the Instituto Nacional de Tecnología Agropecuaria (INTA) in Aristóbulo del Valle, Misiones, Argentina. All sample collection was conducted by a licensed veterinarian. Sample collection protocols were approved by the Ministerio de Ecología y Recursos Naturales Renovables (MEyRNR) in accordance with Law No 47, Regulatory Decree No 474/02, and Resolution No 509/07 of Misiones Province. Collection permits were granted through the Directorate of Ecology and Environmental Quality under Provision No 033, which relates to the handling of animals and collection of samples.

Each paca was captured using a net and manually held in position. Blood and buccal swabs samples were taken after the animal was anesthetized using 6.5 mg/kg Zelazol® (Fort Dodge, Tiletamine-Solazepan). For each paca, 3 mL of blood was extracted from the radial vein and collected in polypropylene tubes anticoagulated with 5% Ethylenediaminetetraacetic Acid (EDTA) and maintained at − 20 °C until subsequent treatments. In addition, three sterile buccal swabs were collected and placed in individually labeled envelopes where they were allowed to air dry prior to storage at room temperature. Finally, six scat samples were collected from their cages, with each sample potentially representing multiple individuals, as two pacas were housed in the enclosure. Scat surfaces were swabbed in triplicate with a cotton-tipped applicator soaked in 1× phosphate buffered saline solution to obtain cells detached from the animal’s intestinal epithelium [28, 29]. Each of these swabs was placed in a 2 mL polypropylene tube and maintained at − 20 °C until DNA extractions were carried out. Scat samples were placed in an individually, labeled plastic bag (18-oz Whirlpak® bag-Nasco, Fort Atkinson, WI) and maintained at − 20 °C, as a backup for DNA extractions.

#### Genetic analyses

DNA was extracted using Cetyl Trimethyl Ammonium Bromide (CTAB) following the corresponding extraction protocol for each sample type [30]. DNA integrity was evaluated in 1% agarose (Invitrogen™) gel and stained with GelRed™ (Biotium).

Of 34 potential primer pairs, 20 that would yield < 300 bp amplicons were selected for cross-amplification testing in paca; 12 developed for guinea pigs (*C. porcellus*; [24]; Table 1) and 8 developed for capybara (*H. hydrochaeris*; [26]; Table 1). All primers sets were synthesized by IDT® (USA) and each primer pair was optimized independently. Amplification assays were conducted in a Perkin Elmer GeneAmp System 9600 thermal cycler (Applied Biosystems) in a 25 μL and 15 μL of final reaction volume for the primers developed for guinea pigs and capybara respectively, containing 1× Green GoTaq® Flexi Buffer, 2.0 mM (guinea pig) and 1.5 mM (capybara) of MgCl_2_, 0.2 μM of each primer (forward and reverse), 0.2 mM of each dNTP, 150 µg/mL Bovine Serum Albumin (BSA), 1U and 0.75U GoTaq® (Promega) DNA Polymerase, and 2 μL of DNA. Negative controls were included in all assays. For those primers used in capybara [26], the published annealing temperatures (T_a_) were used as a starting point in the cross-amplification tests with paca (Table 2), but were adjusted to maximize amplification efficiency.

**Table 1:** Details of the 20 microsatellite primer pairs tested for cross-amplification in paca

ID	Sequence (5′ to 3′)	Repeat motif	Ta (ºC)	Size range (bp)	References
CUY5	F: GGCCAAAGCAGGAATGTCTA	CA	55	141–163	Aviles et al. [24]
	R: TAGGGCAAGCATTGATGATG				
CUY6*	F: TGGCTTGCTTTCTCTTTGGT	CA	55	158–168	
	R: CTGTGCTCAGCATTGCATTT				
CUY7	F: GATGCAGTGCAGAGGAGTCA	CA	55	183–197	
	R: TGTGTGGTTTTGTGTGTGAGG				
CUY8*	F: TGATTGCACCTGAGAAGTGG	TC	55	181–217	
	R: CCAAGTGTTCTTGGTGCTTG				
CUY9	F: GCTGGAATGCAAGACAAGC	GT	55	116–130	
	R: TGAGTTTTCAGCTGTGATGAGT				
CUY10*	F: TTCCAAGCATTTCAGAAAACA	GT	55	106–128	
	R: TGACTTCCCAACCAAGGAAA				
CUY17	F: TGATGGACAATATACTGGGAACC	TC	55	152–170	
	R: TAGCATGCATGAAGCCCTAA				
CUY18*	F: TGTCACTTCTCACTCCACCA	CA	55	176–214	
	R: TCCCAAACCTCTTGTTTGCT				
CUY22	F: CGAACATGCCAAGCAGATTA	TC	55	206–232	
	R: ACACCAGTTCCTTGCCACAT				
CAVY2	F: GGCCATTATGCCCCCCAAC	AC	55	124–154	
	R: AGCTGCTCCTTGTGCTGTAG				
CAVY11*	F: CCGTGCTTTTCCTGTCTTTG	CT	55	140–180	
	R: TGGACCCCAATCTGACATAG				
CAVY12	F: AGAATGCCTTTGGGACTGG	AG	55	143–187	
	R: AGATCTTGCCTCTGCACTTG				
CAPY1*	F: GGAATTCCAAAAGACAACAGTTA	(GT)12	59	185	Herrera et al. [26]
	R: TCTCTCCTCAAAACAACAACAGA				
CAPY4	F: ACACAGGTGCATTTGGCATA	(GT)12	60	190–192	
	R: ATGAGGATGTGGCAGAAAGG				
CAPY6	F: ATGGGGACTCCAGCAAGTTA	(GT)5AT-(GT)7	56	289	
	R: AGATACATGCCTTCCCCAAA				
CAPY7*	F: CCTCAACATCCGTTTTCCTC	(GT)16	60	272–278	
	R: CCCAAGGGTTGAAACACAGT				
CAPY9	F: TGCCATTCTTGTGAAAGGTG	(GT)17	60	152–162	
	R: TGCCCGTTTCAGTGTGTACT				
CAPY10	F: CAAGGCTTCTGCTCACTCATT	(GCT)9	56	182	
	R: TGAGGCATGCAAGAGCAA				
CAPY12	F TGGGTGCCAGAATGCATAGTC	(GT)10	61	195–197	
	R: GCATTGCCACCCCTACCTTA				
CAPY24	F: TGCAGGGAGCACTTTATCCA	(GT)7-TT-(GT)8	54	145–157	
	R: CAAGCTGGGCACAAAAAGGA				

The 12 primer pairs developed for guinea pigs (Aviles et al. [24]) and the 8 primer pairs developed for capybara (Herrera et al. [26]) are detailed in the table. Reported for each primer are the identification (ID), the forward primer sequence (F), the reverse primer sequence (R), the published annealing temperature (Ta), the published expected fragment size (bp), and original reference for these primers. An “*” highlights the seven of heterologous primer pairs that could not be consistently scored and interpreted. The repeat motif numbers were not reported in Aviles et al. [24]

**Table 2:** Summary of the PCR profiles for cross-amplification of microsatellites in paca using 20 heterologous primer pairs developed for guinea pigs and capybara

PCR step	Guinea pig	Capybara			
	All	CAPY1	CAPY9 and CAPY24	CAPY 4, CAPY6, CAPY7, and CAPY10	CAPY12
Initial denaturation	95 °C for 5 min	95 °C for 5 min	–	–	–
# of cycles	34	34	–	–-	–
Denaturation	95 °C for 40 s	94 °C for 30 s	–		–
Annealing	55 °C for 30 s	53 °C for 30 s	54 °C for 30 s	55 °C for 30 s	57 °C for 30 s
Elongation	72 °C for 40 s	72 °C for 40 s	–	–	–
Final extension	72 °C for 5 min	72 °C for 30 min	–	–	–
Maintenance	4 °C	4 °C	–	–	–

All guinea pig primers n = 12 (CUY5, CUY6, CUY7, CUY8, CUY9, CUY10, CUY17, CUY18, CUY22, CAVY2, CAVY11, and CAVY12; Aviles et al. [24] had the same PCR protocol. While capybara primers n = 8 (CAPY1, CAPY4, CAPY6, CAPY7, CAPY9, CAPY10, CAPY12 and CAPY24; Herrera et al. [26]) had similar conditions across the majority of the PCR profile, indicated by “–” in the table, they varied in annealing temperature

Amplification success was initially verified in 3% agarose gels using the previously described technique, except for 50 min at 5 V/cm. For allele discrimination, amplicons were analyzed using 6% vertical electrophoresis denaturing polyacrylamide gels (acryl:bisacrylamide 32:2) including a 25 bp DNA Ladder (Invitrogen™) as a molecular marker in 0.5 × TBE for 90 min at 60 W. Gels were stained with Silver Sequence™ Staining Reagent kit (Promega) and digitally documented with Kodak EasyShare Z7590 camera.

To confirm successful cross-amplification of microsatellite regions in paca, three amplicons, obtained from blood samples, were randomly selected to be sequenced through the Sanger methodology at Macrogen, Inc. Korea.

### Results

Thirteen (65.0%) of heterologous primers pairs amplified DNA extracted from blood (100% of samples), saliva (46.2% of samples), and scat (20.5% of samples), while seven (35.0%) of heterologous primers pairs could not be consistently scored and interpreted (Table 1). Seven (53.9%) of the microsatellite primers pairs that successfully cross-amplified in paca were from the twelve developed for guinea pigs (CUY5, CUY7, CUY9, CUY17, CUY22, CAVY2, and CAVY12; Table 1). The remaining six (46.2%) successful primers were from the eight described for capybaras (CAPY4, CAPY6, CAPY9, CAPY10, CAPY12, and CAPY24; Table 1). The efficiency of the cross-amplification in paca was verified with capillary electrophoresis on an ABI3130XL (Macrogen, Inc) that compared the expected repeat motif for each primer pair in three randomly selected primers (CUY7, CUY9, and CAPY24; Table 1); however, sequences could not be uploaded to GenBank due to the fact all were < 200 bp in size or below the minimum size required by this service.

Thirteen (65.0%) of the tested loci in paca produced amplicon size ranges similar to those in the two species (guinea pig and capybara) for which the primers were originally developed (Table 3). Three primers (CAVY2, CAVY12, and CAPY9) had amplicons that fell within the published ranges for guinea pig and capybara. Eight primers (CUY7, CUY9, CUY17, CAPY4, CAPY6, CAPY10, CAPY12 and CAPY24) had amplicons that showed light shifts (± 1 to 49 bp) from the expected size ranges. Two primers (CUY5 and CUY22) had amplicons that showed larger shifts (± 43–130 bp) from the published size ranges. In addition, while two primers (CAPY6 and CAPY10) were published as homozygous in capybara, both were heterozygous in paca. All 13 (100%) of these amplified loci were polymorphic (range: 2–6 loci), showing different sized loci (Table 3).

**Table 3:** Allelic details for the 13 microsatellite primer pairs that successfully cross-amplified in paca

Primers	B1	B2	B3	S1	S2	S3	Scat 1	Scat 2	Scat 3	Scat 4	Scat 5	Scat 6	Total
Guinea pig													
CUY5	204; 204	204; 202	204; 202	204; 204	204; 204	204; 202	–	–		206; 206	–	–	3
CUY7	194; 186	194; 186	194; 186	194; 186	194; 186	194; 186	194; 186	198; 182	192; 192	192; 192	–	192; 188	6
CUY9	138; 138	144; 142	142; 140	138; 138	144; 142	142; 140	140; 140	–	–	–	138; 138	–-	4
CUY17	174; 162	174; 164	172; 162	–	–	–	–	–	–	–	–	–-	4
CUY22	102; 102	102; 102	102; 100	–	–	–	–	–	–	–	–	–-	2
CAVY2	146; 142	146; 142	146; 142	146; 142	–	–	–	–	–	–	–	–	2
CAVY12	160; 158	160; 158	160; 158	160; 158	–	–	–	–	–	162; 160	–	–	3
Capybara											–-	–-	
CAPY4	172; 172	172; 172	172; 172	172; 172	170; 168	170; 170	170; 168	170; 168	–	–	–	–	3
CAPY6	266; 246	266; 246	268; 248	–	–	–	–	–	–	–	–	–	4
CAPY9	154; 154	154; 154	156; 156	–	–	–	–	–	–	–	–	–	2
CAPY10	216; 208	216; 208	216; 208	216; 208	–	–	208; 206	–	–	–	–	–	3
CAPY12	246; 198	246; 198	242; 194	–	–	–	–	–	–	–	–	–	4
CAPY24	120; 116	122; 118	120; 116	120; 116	122; 118	120; 116	124; 118	120; 116	–	–	118; 114	118; 116	6

Specific sizes (bp) and total number of alleles (Total) are reported for each primer across the three blood samples (BI, BII, and BIII), three saliva samples (S1, S2, and S3), and six scat samples (Scat1, Scat2, Scat3, Scat4, Scat5 and Scat6). The thirteen pair of primers were polymorphic. No data is indicated by “–”

## Discussion

Using heterologous primers, we identified 13 microsatellite primers pairs that will facilitate future studies focused on identifying individual paca from scat or other noninvasive, fragmented DNA sources. Gaining genetic information from noninvasive samples has increased the amount of information that can be obtained [31], especially in the field of conservation. It also allows insight into species distribution [32, 33], habitat preferences and requirements [34, 35], and diet [36, 37]. Expanding this to the individual-level provides knowledge on gender identification and population ratios [38, 39], species abundance [40, 41], and population density [42, 43]. Together, these genetic data can be used to investigate broader population-level questions including rates of gene flow [44, 45], genetic diversity [46, 47], and phylogeographic studies [35, 48].

To confirm successful cross-amplification of microsatellite primers in paca, two different approaches were used. First, the Sanger sequencing results of three amplicons obtained using CUY7, CUY9, and CAPY24 showed that sequences matched the expected repeat motif published for microsatellite primers developed for guinea pigs and capybara. For paca, guinea pig primers CUY7 and CUY9 shared the repeat motif sequence (CA) and (GT), respectively. With capybara primer CAPY24, the sequence showed the same (GT) repeat motif, with the exception that paca lacked the (TT) interruption. Second, amplification results for blood and saliva were compared within individuals for consistency. Again, in all cases, allele sizes matched. It was not possible to compare similarities between blood and saliva with scat, as the latter represented a mixed sample from multiple pacas versus single individual. Therefore, the scat samples often represented different allelic combinations compared to the blood and saliva (Table 3).

Not surprisingly, amplification was not possible in all scat samples. This variation can be attributed to several factors including the presence of inhibitors in the extracted DNA, low concentrations of extracted DNA, and extremely fragmented or degraded DNA [31, 40, 49–52]. All of the primers (n = 13 or 100%) tested in this study were polymorphic, which permits individuals in a population to be differentiated. It is suspected these primers would be even more informative in a free-ranging paca study, as those populations would likely have higher genetic variability compared to captive populations [53–55]. The low number of founders in the used captive population would suggest it likely possesses some levels of homozygosity (e.g., revealed by monomorphic microsatellites) and low genetic variability [56]. However, we cannot rule out that the low level of STRs variability observed in some primers might be a result of allelic dropout because the heterologous status of the primers used or allele fixation in the population due to inbreeding [57].

Additional work is needed in free-ranging paca populations to determine the number of microsatellite loci needed to distinguish among individuals, a number that varies depending on the population’s genetic variability and the expected loci heterozygosity [49]. The number of loci needed must fit a balance between achieving the lowest probability of individual identity, while minimizing costs and using the lowest number of loci needed [58]. It is clear from these data on captive paca that the selection of loci that allows individuals to be identified with certainty can be a difficult task in small populations that have suffered bottlenecks in genetic variability due to genetic drift [31]. The 13 heterologous primers confirmed to successfully cross-amplify in paca are the first step to understanding genetic variability in free-ranging paca, which can form the foundation for applied management strategies for this large rodent that is favored by predators and humans alike.

## Limitations

- The number of individuals used in the sampling was low and would ideally be expanded; however, INTA captive breeding centre limited the number authorized for use in this study.
- The low number of founders in the INTA captive population likely limited genetic variability seen among the 13 proposed primer pairs.
- Testing of primer cross-amplification used exclusively captive individuals due to difficulty in obtaining invasive sample from free-ranging individuals.

## Data Availability

The datasets generated and/or analysed during the current study are not publicly available, as the sequences output sizes were < 200 bp, which is lower than the minimum length accepted by GenBank. Nevertheless, the datasets are available from the corresponding author on reasonable request.

## Abbreviations

BSA: Bovine serum albumin
CTAB: Cetyl trimethyl ammonium bromide
DNA: Deoxyribonucleic acid
EDTA: Ethylenediaminetetraacetic acid
INTA: Instituto Nacional de Tecnología Agropecuaria
MEyRNR: Ministerio de Ecología y Recursos Naturales Renovables
SSR: Single sequence repeats
STR: Short tandem repeats
TBE: Tris borate EDTA
UV: Ultraviolet
